# The GRONORUN 2 study: effectiveness of a preconditioning program on preventing running related injuries in novice runners. The design of a randomized controlled trial

**DOI:** 10.1186/1471-2474-11-196

**Published:** 2010-09-01

**Authors:** Steef W Bredeweg, Sjouke Zijlstra, Ida Buist

**Affiliations:** 1 University Center of Sport, Exercise and Health, University Medical Center Groningen, Hanzeplein 1, 9700 RB Groningen, The Netherlands; 2 Center for Sports Medicine, University Medical Center Groningen, Hanzeplein 1, 9700 RB Groningen, The Netherlands

## Abstract

**Background:**

Distance running is a popular recreational exercise. It is a beneficial activity for health and well being. However, running may also cause injuries, especially of the lower extremities. In literature there is no agreement what intrinsic and extrinsic factors cause running related injuries (RRIs). In theory, most RRIs are elicited by training errors, this too much, too soon. In a preconditioning program runners can adapt more gradually to the high mechanical loads of running and will be less susceptible to RRIs. In this study the effectiveness of a 4-week preconditioning program on the incidence of RRIs in novice runners prior to a training program will be studied.

**Methods/Design:**

The GRONORUN 2 (Groningen Novice Running) study is a two arm randomized controlled trial studying the effect of a 4-week preconditioning (PRECON) program in a group of novice runners. All participants wanted to train for the recreational Groningen 4-Mile running event. The PRECON group started a 4-week preconditioning program with walking and hopping exercises 4 weeks before the start of the training program. The control (CON) and PRECON group started a frequently used 9-week training program in preparation for the Groningen 4-Mile running event.

During the follow up period participants registered their running exposure, other sporting activities and running related injuries in an Internet based running log. The primary outcome measure was the number of RRIs. RRI was defined as a musculoskeletal ailment or complaint of the lower extremities or back causing a restriction on running for at least three training sessions.

**Discussion:**

The GRONORUN 2 study will add important information to the existing running science. The concept of preconditioning is easy to implement in existing training programs and will hopefully prevent RRIs especially in novice runners.

**Trial registration:**

The Netherlands National Trial Register NTR1906. The NTR is part of the WHO Primary Registries.

## Background

Running is a popular activity and can be practised everywhere. The health benefits are substantial but runners get injured regularly. The incidence of running related injuries (RRIs) is high. Various studies in different populations reported rates of RRIs ranging from 19-79% [1-9]. RRIs are often located in the lower extremities with knee and lower leg mostly affected [3,6,7,10-12]. There is no agreement on the cause of RRIs. In the current literature, possible intrinsic and extrinsic risk factors are identified, but there is still no exact cause for an RRI. Van Mechelen [13] and van Gent et al [9] proposed risk factors that have been significantly related to RRI; excessive weekly running distance, previous injury, lack of running experience and competitive running.

Clinical studies showed that over 60% of RRIs could be attributed to training errors [14]. Hreljac [14] stated that all overuse running injuries are the result of training errors. From this point of view a RRI is a disturbance between the external load applied to the body and the injury threshold of a biological structure of the body. In this dose-response relationship there are four components applicable to the novice runner [14]. The first component is the current status of the musculoskeletal system of the novice runner. The second component is the type of applied stress (i.e. running). Thirdly, the frequency, intensity and duration of the applied stress (i.e. running) and finally, the adaptation and recovery times between running sessions are major determinants of this dose relationship.

Under normal circumstances the musculoskeletal system adapts to the level of stress placed upon it [14-16]. When an optimal level of stress is applied to the musculoskeletal system, along with an adequate recovery time, the musculoskeletal system will increase in strength. On the other hand, when the applied stress is too high or the recovery time is too short the tissue of the musculoskeletal system will be weakened and the likelihood of sustaining a subsequent overuse injury is high [14,16,17]. Mechanical load (i.e. running) applied to the human body can cause a physiological or pathological adaptation to this mechanical loading, resulting in respectively a training effect or overuse injury [17].

The musculoskeletal system of the novice runner is normally not adapted to the repetitive and relatively high impact forces of running because novice runners are frequently physically inactive before they start to run [10,18]. In most regular running programs for novice runners the biomechanical load is high from the start of the program in terms of frequency, intensity and duration.

The first GRONORUN study [7,19] showed that previous sports participation without axial loading was an important predictor for RRIs in novice runners. From this knowledge a strategy can be chosen to strengthen the lower extremities to achieve a positive physiological adaptation of the musculoskeletal system before starting a training program for novice runners. The applied external load of this so called preconditioning program will stress the lower extremities and as a result the lower extremities will positively adapt to the applied stress. In this way there is a stepwise transition of biomechanical load which makes it easier for the musculoskeletal system of the lower extremities to withstand the demands of running. Other studies in athletes and military populations [20-23,23] showed a positive effect of a preconditioning program on the incidence of sports and overuse injuries in different populations.

In a preconditioning program for running, the program needs to load the musculoskeletal system in a sport-specific way. Therefore, in this randomized controlled trial a preconditioning program with walking and hopping prior to the training program for novice runners will be studied. We hypothesize that the novice runner can adapt more gradually to the external impact forces of running with a preconditioning program prior to the training program and will be less susceptible to RRIs.

## Methods/design

The GROningen NOvice RUNing 2 (GRONORUN 2) study is a randomized controlled trial with a 13-week follow-up. Participants were randomized into two groups: an active control (CON) group and an intervention (PRECON) group. The PRECON group will receive a 4-week preconditioning program prior to the start of the training program. Recruitment of participants for the GRONORUN 2 study took place in the period April - June 2008 and data collection started in July 2008. The study design, procedures and informed consent procedure were approved by the Medical Ethics Committee of the University Medical Center Groningen (No. 2007.217). All participants provided written informed consent. Guidelines were followed according to the Consort Statement [24].

### Study population

In the period April - June 2008, participants who were willing to start a "beginners 9-week program" in preparation for the Groningen 4-Mile running event were recruited with advertisements in local media in the northern part of the Netherlands. For this study participants were not obliged to participate in the Groningen 4-Mile running event. The Groningen 4-Mile running event is a popular annual recreational running event that takes place in October. After initial registration, potential participants were sent written information about the study along with a baseline questionnaire and invitation for an initial interview in the Center for Sports Medicine at the University Medical Center Groningen (UMCG), The Netherlands.

### Inclusion & exclusion criteria

Healthy subjects between 18 and 65 years of age who had no injury of lower extremities or lower back in the last three months prior to inclusion, who had not been running on a regular basis in the previous twelve months who were willing to start a beginners program were eligible for inclusion in the study. Potential participants were excluded to the study if there were absolute contraindications for vigorous physical activities according to the American College of Sports Medicine [25] or in case of unwillingness to keep a running log.

### Sample size

A power calculation was carried out for the main outcome variable, i.e. running related injury (RRI), using a logistic rank survival power analysis. As stated before, the incidence of RRIs varies between 19-79%.

A reduction of 25% on the incidence of RRIs in the PRECON group is considered clinically significant and relevant. The expected incidence of RRIs is 40% [4,10]. With a hypothesized 25% reduction of RRIs in the PRECON group compared to the control group, a total of 360 runners (2 × 180) is needed for a power of 80% and an alpha of 0.05. Assuming an attrition of 15% in the intervention period and follow up period, a total of 414 (2 × 207) novice runners are needed to detect an effect of the PRECON intervention.

### Baseline questionnaires

All participants filled in an online questionnaire before baseline measurements were taken. In case potential participants had no access to the internet a questionnaire was sent by mail. Demographic and anthropometric variables that were collected were age, gender, body weight and length. Conditions related to risk factors for cardiovascular diseases were assessed using a series of questions according to the American College of Sports Medicine [25]. Past musculoskeletal complaints of the lower extremities and back were assessed by questions on the anatomical site and the number of days lost to work and/or sporting activities. When a musculoskeletal complaint was caused by a sporting activity it was registered as a previous sports injury. When the musculoskeletal complaint was caused by running in the past it was registered as a previous running injury. Sports participation was measured by asking whether someone was participating in sports in the past twelve months (yes/no), type of sport and mean hours of sport participation per sport a week. Furthermore a question on running experience in the past ("did you ever structurally run before") was added to assess the novelty to running.

After receiving the complete questionnaire potential participants were invited for an initial interview by an experienced sports physician at the Sportsmedicine Center of the University Medical Center Groningen. The purpose of the initial interview was to screen for cardiovascular diseases and abnormalities of lower limb and to ensure that the participants were eligible and were adequately informed about the study before signing informed consent for the GRONORUN 2 study.

### Baseline orthopaedic measurements

Hip function was measured by using a universal goniometer with arm length 30 cm from axis to tip. The internal and external range of motion of the hip was assessed with the participant supine and the tested hip and knee flexed to 90°. Knee flexion and extension ranges of motion were assessed with the participant in supine position. The goniometer was placed on the lateral aspect of the knee, with the axis of the goniometer in line with the greater trochanter and the lateral malleolus. Ankle plantar flexion and dorsi flexion were measured both with the knee fully extended and flexed to 90°. One arm of the goniometer was aligned with the fibular bone and the other with the plantar surface of the foot. Furthermore, the navicular drop was assessed by measuring the change in the height of the navicular tuberosity between a participant sitting with the subtalar joint in neutral position and standing, weight bearing with the subtalar joint in relaxed stance, as described by Brody [26]. The navicular drop is a valid method to indicate the amount of foot pronation [27]. Intratester and intertester reliability of this technique is ranging from .73 to .96 [28]. Measurements were made twice for each foot, with results being averaged. These measurements were identical to the GRONORUN 1 study [19].

### Randomization

After baseline measurements and informed consent, participants were randomly assigned to the CON or the PRECON training program.

To ensure that both groups were equal in terms of injury risk, a stratified randomization was performed based on three variables; current sporting activities, previous injuries and gender. Based on current sporting activities, there were three categories of novice runners. The first category consisted of novice runners who already were participating in a sport in which axial load i.e. running, walking or jumping, was integrated. The second category was formed by novice runners who already were participating in sporting activities without axial load, like swimming and cycling. The third and last category was formed by novice runners who did not participate in any sporting activities at baseline measurements.

In a study by Macera [1], a 74% increased risk was found in runners with a positive history of previous injuries. In this study, previous musculoskeletal complaints with an impact of activities of daily life, work or sporting activities were defined as a previous injury. Since it is not clear whether the high rate of re-injury is caused by incomplete healing of a previous injury or a biomechanical problem, a differentiation in time is made. A distinction can be made between no previous injury, injuries sustained in the last 12 months before baseline measurements and injuries sustained more than 12 months before baseline measurements. The participants were also stratified for gender because men and women differ in incidence of RRIs and localisation of these injuries [6,9]. In total eighteen strata were formed by gender, previous injury (no injuries, injury 3 till 12 months ago and injuries longer then 12 months ago) and sporting activities (no, with axial load and without axial load). From each stratum, participants were randomly allocated to intervention or control group by drawing a sealed opaque envelope. Each stratum box contained equal numbers of control and intervention envelopes.

### Participant flow

**Figure 1:**
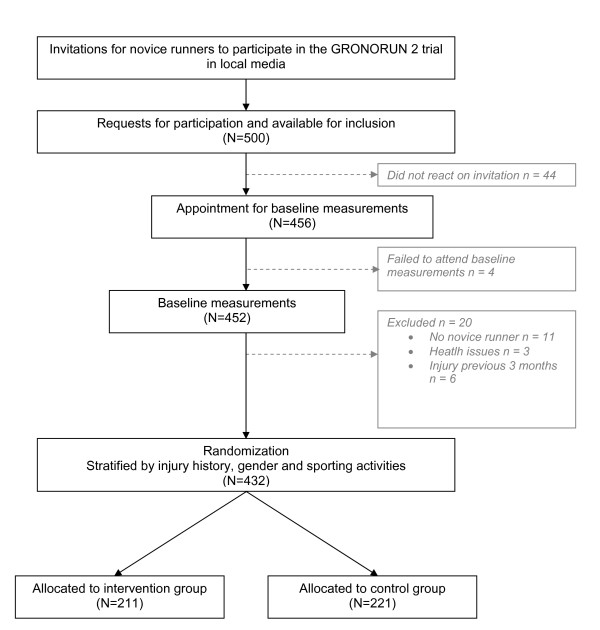
**Flow chart of the GRONORUN 2 study**.

The study design and participants flow are shown in Figure 1. A total of 500 people were interested to participate in the GRONORUN 2 study and responded to the call for novice runners. To all of those who reacted on the advertisements, an information brochure in which the study protocol was clearly described, a baseline questionnaire and an appointment at the UMCG was given. Forty four did not confirm their appointment for the initial interview nor filled in the baseline questionnaire. Of those who confirmed the appointment for the initial interview and filled in the questionnaire (n = 456), four failed to attend the initial interview. Eventually, of the 452 persons who visited the UMCG for an initial interview, 20 were excluded. Reasons for exclusion were: already participating in running (n = 11), musculoskeletal injury of lower extremities or back at baseline (n = 6) and contraindications for vigorous physical activity (n = 3). After baseline measurements and stratification, 432 participants were randomly assigned to the intervention group (n = 211) and to the control group (n = 221).

### Movie: Correct hopping technique

#### Training program

##### 4-week preconditioning program

The PRECON group received a 4-week individual preconditioning training program (Table 1). This program gradually increases biomechanical load on the lower extremities with walking and hopping sessions. Participants were instructed to walk briskly on their running shoes three times a week. During two of the three walking sessions per week, participants carried out hopping exercises. After every five minutes of walking a session of hopping was carried out. In approximately half an hour six sessions of hopping were carried out. The number of hops as well as the weekly walking distance increased gradually. The PRECON group received verbal information about the correct hopping technique at the initial interview and there was a video instruction on the personalized environment of the internet based training log of the PRECON group.

**Table 1:** The 4-week preconditioning (PRECON) program with walking and hopping

	PRECON training 1			PRECON training 2			PRECON training 3	Total	
	**Walk** **(min)**	**Hop**	***(rep.)***	**Walk** **(min)**	**Hop**	***(rep.)***	**Walk** **(min)**	**Walk** **(min)**	**Hop**

week 1	5	50	*(6)*	5	60	*(6)*	30	**90** **[7500 BW]**	**660** **[2310 BW]**
week 2	5	60	*(6)*	5	70	*(6)*	45	**105** **[8750 BW]**	**780** **[2730 BW]**
week 3	5	70	*(6)*	5	80	*(6)*	60	**120** **[10000 BW]**	**900** **[3150 BW]**
week 4	5	80	*(6)*	5	90	*(6)*	60	**120** **[10000 BW]**	**1020** **[3570 BW]**
week 5	Start of the 9-week training program								

The content of the PRECON training program is expressed in minutes of walking (Walk) and the number of hopping in place (Hop) and the amount of repetitions (rep.) of walking and hopping. The right column contains total minutes of walking and total performed hops each week. In the last two columns the additional load of walking and hopping each week is expressed between brackets [] in bodyweight (BW)

The correct technique of hopping in place was a relaxed standing position with a distance of approximately 30 cm between the left and right foot with both hands in the sides. Then small jumps in place were performed with the forefeet almost keeping contact with the ground (Additional file 1). During the 4 weeks of the preconditioning program of the PRECON group the CON group was instructed to do their normal exercise routine and sporting activities if applicable.

In the first training week, in which both groups are starting to run, the theoretical extra biomechanical load of running (10 km/h; total 30 min, impact 2.0 bodyweight (BW) per landing) and walking (5 km/h, total 30 min, impact 1.0 BW per landing) is approximately 12,500 extra BW. Walking is a low cyclic impact force activity and hopping is a high cyclic impact force activity. Both activities stress the body in a cyclic way, especially the lower extremities, so that the body has to positively adapt to the biomechanical stimuli.

**Figure 2:**
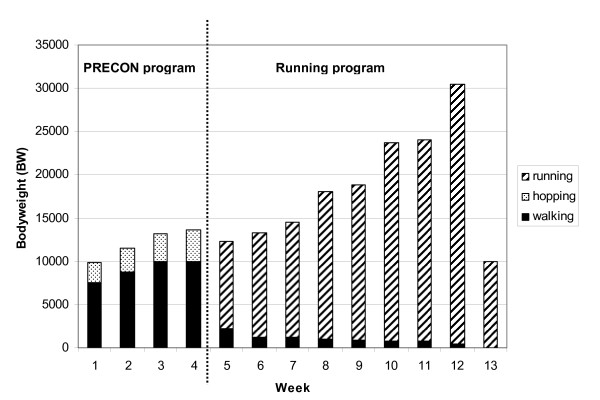
**Biomechanical load for the PRECON (week 1-13) and the CON (week 5-13) group**. The load of walking, hopping and running is expressed in bodyweight (BW) per week. Week 1-4 was the intervention period of the PRECON group. From week 5 both groups followed the same training program.

In the first week of the PRECON program the extra biomechanical load of walking (5 km/h, total 90 min, impact 1.0 BW per landing) and hopping (660 hops, impact per hop 3.5 BW per landing) was approximately 9810 extra BW. In week 2 there is 11480 extra BW, in week 3 13150 BW and in week 4 13570 BW (Figure 2).

##### The 9-week training program

Nine weeks before the Groningen 4-Mile running event all participants were instructed to start their 9-week training program (Table 2). Participants of the CON and PRECON group received the same general written and oral information on intensity of running and on warming up and cooling down. Participants were instructed to walk briskly for 5 minutes as a warm up, and 5 minutes as cool down. Given that the best available evidence indicates that stretching before or after exercise does not prevent muscle soreness or injury [29], participants were instructed not to perform stretching exercises before, during or after the training sessions.

**Table 2:** The 9-week training program for both groups

	Training 1			Training 2			Training 3			Total	
	**run**	***walk***	**(rep.)**	**run**	***Walk***	**(rep.)**	**run**	***walk***	**(rep.)**	**Run**	***walk***

week 1	**1**	*1*	(10)	**1**	*1*	(10)	**1**	*1*	(10)	**30**	*27*
week 2	**2**	*1*	(6)	**2**	*1*	(6)	**2**	1	(6)	**36**	*15*
week 3	**4**	*2*	(3)	**4**	*2*	(4)	**4**	*2*	(3)	**40**	*14*
week 4	**6**	*2*	(3)	**5**	*2*	(3)	**6**	*2*	(3)	**51**	*12*
week 5	**6**	*2*	(3)	**9**	*2*	(2)	**6**	*2*	(3)	**54**	*10*
week 6	**8**	*2*	(3)	**15**	*0*	(1)	**15**	*5*	(2)	**69**	*9*
week 7	**10**	*2*	(2)	**15**	*5*	(2)	**10**	*2*	(2)	**70**	*9*
week 8	**30**	*0*	(1)	**15**	*5*	(2)	**30**	*0*	(1)	**90**	*5*
week 9	**30**	*0*	(1)				**The Groningen running 4-Mile event**			**30**	*0*

The content of each training session is expressed in minutes of running (run), minutes of walking between the running sessions (walk) and number of repetitions (rep.). The last two columns contains the total minutes of running and walking for each week.

The frequency of running was equal in both groups. Each training week except the last week i.e. the week of the Groningen 4-Mile running event, consisted of three training sessions represented by a combination of running and walking. Participants were encouraged to run on Monday, Wednesday and Saturday or on Tuesday, Thursday and Sunday. Runners were advised to run at a comfortable pace at which they could converse without breathlessness. Both groups trained individually, without a trainer on a self-chosen course.

### Outcome measures

The primary outcome of the GRONORUN trial was the number of RRIs in both groups. A runner could only have one RRI. Definition of a RRI in this trial was; running related musculoskeletal ailment of the lower extremities or back, causing a restriction of running for at least one week, i.e. three consecutive training sessions.

Information on RRIs and exposure data was collected using an internet based running log. Each of the participants received a study number and a password to enter a personal environment of the web based training log. After each training week participants had to fill in their running activities, other sport activities and injuries.

Per training session the total minutes of running, total minutes of walking and injuries were registered. Data on injuries were collected by registering anatomical site of the body and severity of pain. Severity of pain was subdivided in pain without limitation (no RRI), pain that caused a restriction of running (scored as an RRI) and pain which made running impossible RRI (scored as an RRI). In case of skipping a training session, the reason (RRI, other injury, motivation, illness or remaining reason) for it was asked. When a "running related injury" was the reason for not training, information on anatomical site and severity was asked. To point out the anatomical site of an injury, a picture of the lower body was shown after reporting a RRI. By clicking on the anatomical site of the RRI, the same spot was appointed in red. When participants did not enter their digital training log after one week, a reminder was send by email automatically. In case of not having access to the Internet, all participants had also a hard copy of the running log.

### Statistical analyses

To evaluate the success of the randomization, baseline characteristics of participants in the CON and PRECON group were compared using 2-tailed t-tests for normally distributed continuous variables. The χ^2 ^statistic was used for discrete variables. To evaluate the effect of the PRECON program on the number of injured runners in both groups, a χ^2^-test was used. The log-rank test is used to compare the Kaplan-Meier curves of the injured runners of the PRECON group and the CON group, analyzing the difference between these two groups in the probability of an RRI at any point in time. All analyses were performed following the "intention to treat" principle. Differences were considered statistically significant at P < .05. All analyses were performed using SPSS version 16.0 (SPSS Inc, Chicago, Ill).

## Discussion

To study the population of novice runners it is important because the main reason for discontinuation (drop out) of a running program is injury [18]. Negative experiences, caused by an injury that occurs while training for a running event, have the potential to significantly affect the future physical activity of each individual. It is also known that (fear of) sustaining an injury is associated with failure to start and maintain a physically active lifestyle [30].

As stated by Yeung [31] there is a need for more well controlled trials to shed light on possible interventions for the prevention of lower limb injuries in runners. Current studies on the effect of interventions for preventing running injuries in recreational runners are scarce. The GRONORUN 1 study [7] showed no effect of a more gradual training program in the novice recreational runners.

In preventive medicine it is important to develop interventions based on the understanding of the etiology and mechanisms of injury and the preventive intervention has to be acceptable, practical and adopted by athletes and sport bodies so that the implementation of the intervention can be successful [32]. The proposed intervention in this RCT is practical, easy to do and therefore has a good chance for success in terms of compliance, efficacy and effectiveness.

Results of this GRONORUN 2 study can be implemented in the existing training program for novice runners and the new preconditioning training program can be implemented on a regional, national and international level. In this way, a more scientific based training program for novice runners can be developed and novice runners will feel safer in starting a running program.

With this study there is also a unique opportunity to start more clinical and preventive studies on overuse running related injuries. The newly gathered information will be transferred into new clinical and preventive studies in the future.

## Competing interests

The authors declare that they have no competing interests.

## Authors' contributions

SWB conceived of the idea, obtained funding for the study and developed the intervention. SWB and IB developed the design of this trial and recruited participants. SWB is the study investigator and wrote the article, SZ was responsible for data acquisition. IB was co applicant of the grant.

All authors read and approved the final manuscript.

## Pre-publication history

The pre-publication history for this paper can be accessed here:

http://www.biomedcentral.com/1471-2474/11/196/prepub

## Supplementary Material

Additional file 1 **Suppl1**. Click here for file
